# “Manganese-induced neurotoxicity: a review of its behavioral consequences and neuroprotective strategies”

**DOI:** 10.1186/s40360-016-0099-0

**Published:** 2016-11-04

**Authors:** Tanara V. Peres, Maria Rosa C. Schettinger, Pan Chen, Fabiano Carvalho, Daiana S. Avila, Aaron B. Bowman, Michael Aschner

**Affiliations:** 1 Department of Molecular Pharmacology, Albert Einstein College of Medicine, Forchheimer, 209, 1300 Morris Park Ave, Bronx, 10461 NY USA; 2 Department of Biochemistry and Molecular Biology, CCNE, Federal University of Santa Maria, Camobi, Santa Maria, Brazil; 3 Laboratório do Grupo de Pesquisa em Bioquímica e Toxicologia em Caenorhabditis elegans (GBToxCe), Universidade Federal do Pampa, Uruguaiana, RS Brazil; 4 Department of Pediatrics, Vanderbilt University Medical Center, Nashville, TN USA

**Keywords:** Manganese, Manganese-transporters, Acetylcholine, Neuroprotection

## Abstract

Manganese (Mn) is an essential heavy metal. However, Mn’s nutritional aspects are paralleled by its role as a neurotoxicant upon excessive exposure. In this review, we covered recent advances in identifying mechanisms of Mn uptake and its molecular actions in the brain as well as promising neuroprotective strategies. The authors focused on reporting findings regarding Mn transport mechanisms, Mn effects on cholinergic system, behavioral alterations induced by Mn exposure and studies of neuroprotective strategies against Mn intoxication. We report that exposure to Mn may arise from environmental sources, occupational settings, food, total parenteral nutrition (TPN), methcathinone drug abuse or even genetic factors, such as mutation in the transporter SLC30A10. Accumulation of Mn occurs mainly in the basal ganglia and leads to a syndrome called manganism, whose symptoms of cognitive dysfunction and motor impairment resemble Parkinson’s disease (PD). Various neurotransmitter systems may be impaired due to Mn, especially dopaminergic, but also cholinergic and GABAergic. Several proteins have been identified to transport Mn, including divalent metal tranporter-1 (DMT-1), SLC30A10, transferrin and ferroportin and allow its accumulation in the central nervous system. Parallel to identification of Mn neurotoxic properties, neuroprotective strategies have been reported, and these include endogenous antioxidants (for instance, vitamin E), plant extracts (complex mixtures containing polyphenols and non-characterized components), iron chelating agents, precursors of glutathione (GSH), and synthetic compounds that can experimentally afford protection against Mn-induced neurotoxicity.

## Background

Manganese (Mn) is a naturally occurring heavy metal present as the fifth most abundant metal in the environment and twelfth most abundant element as a whole. Mn is essential for humans and animals and daily requirements are commonly met by an adequate diet. Legumes, rice, nuts and whole grains contain the highest levels of the metal. Mn is also found in seafood, seeds, chocolate, tea, leafy green vegetables, spices, soybean, and some fruits such as pineapple and acai. An overview of Mn content in common Mn-rich foods can be found in Table 1. The recommended daily intake of Mn for adult men is 2.3 and 1.8 mg/day for adult women [1]. For children, these values vary with age and are shown in Table 2. For ages 0 to 6 months the Institute of Medicine’s Dietary Reference Intake for Mn cites an adequate intake (AI) that reflects the observed mean Mn intake from human milk. In an earlier study, total Mn secretion in human milk was estimated to be 1.9 μg/day over the first 3 months and 1.6 μg/day over the second 3 months [2]. Based on these values, the AI is set according to average milk volume consumption (0.78 L/day). At ages 7 to 12 months, with the introduction of complementary foods, AI is increased. For ages 1 through 18 years, the AI is based on median Mn intake data obtained from the Food and Drug Administration Total Diet Study. The Dietary Reference Intake also lists 9–11 mg/day Mn as the upper tolerable limit likely to pose no risk of adverse health effects for adults, and 2–6 mg/day Mn for children, depending on the age. Only a small percentage of these amounts are absorbed from the intestine, since the gut tightly controls body Mn load and the metal is rapidly and efficiently excreted via bile as long as no hepatic disease takes place [3, 4].

**Table 1:** An overview of manganese (Mn) content in food and drinks

Food	Country of origin	Mn content (±SD)	Reference
Apple	Italy and Bucovat Nigeria	0.95 μg/g 0.53 ppm	[212] [213]
Beef meat	Xangai, China	0.848 ± 1.10 μg/g	[214]
Cabbage	Egypt	37.80 ± 1.20 μg/g	[215]
Cherry	Bulgaria Bucovat	0.65 μg/g 0.80 μg/g	[212]
Chicken meat	Xangai, China	0.561 ± 4.18 μg/g	[214]
Chinese chive	South Korea	1.17 ± 0.04 μg/g	[216]
Coffee	Bosnia and Herzegovina Brazil Lebanon Poland	5.02 ± 0.52 μg/g 4.97 ± 0.10 μg/g 6.19 ± 0.02 μg/g 6.28 ± 1.01 μg/g	[217]
Duck meat	Xangai, China	0.54 ± 0.23 μg/g	[214]
Freshwater fish	Xangai, China	0.47 to 0.96 μg/g	[214]
Garlic	Kosovo	0.012 to 1.14 μg/g	[218]
Grape	Egypt Nigeria	0.75 μg/g 0.08 ppm	[212] [213]
Infant formula			
Cow-based Soy-based	USA	30–50 μg/L 200–300 μg/L	[220]
Lemon	Nigeria	0.23 ppm	[213]
Lettuce	Egypt	20.0 ± 1.0 μg/g	[215]
Marine mussels *Mytilus edulis and* *Perna viridis*	England, USA, Sweden, Scotland, Canada, China.Hong Kong, China, Malasya,	10 to 100 μg/g 10 to 150 μg/g	[221]
Melon	Periam Turkey	0.65 μg/g 0.85 μg/g	[212]
Milk			[222]
Fresh or processed Human milk	Pakistan –	0.0215 ± 0.01 or 0.0166 ± 0.01 ppm 3 to 10 μg/L	[220]
Mineral water	Egypt	2.35 ± 0.16 ɳg/mL	[215]
Onion	Kosovo	0.002 to 1.29 μg/g	[219]
Orange	Nigeria	0.45 ppm	[213]
Pears	Italy Bucovat	0.40 μg/g 0.90 μg/g	[212]
Pineapple	Nigeria	15.00 ppm	[213]
Plums	Chile Romania	0.95 μg/g 1.05 μg/g	[212]
Pork meat	Xangai, China	0.602 ± 0.697 μg/g	[214]
Potatoes (white, red, orange-fleshed)	Canary Islands, Spain	2.71 ± 2.22, 2.57 ± 1.69, 1.74 ± 0.14 μg/g	[223]
Radish	Kosovo	0.038 to 1.33 μg/g	[219]
Rice	Australia	24.4 μg/g	[224]
River water	Egypt	2.16 ± 0.16 ɳg/mL	[215]
Seawater fish	Xangai, China	0.437 to 0.953 μg/g	[214]
Shellfish	Xangai, China	0.437 to 18.15 μg/g	[214]
Soybean			
Non-transgenic samples	Brazil	16.4 to 24.7 μg/g	[224]
Transgenic samples Soy extracts	Japan Canada	18.2 to 44.3 μg/g 16.5 ± 8.6 μg/g	[225] [226]
Spinach	Egypt Kosovo South Korea	24.60 ± 1.10 μg/g 0.006 to 1.25 μg/g 3.69 ± 0.0001 μg/g	[215] [218] [216]
Strawberry	Belgium Romania	3.65 μg/g 2.15 μg/g	[212]
Tap water	Egypt	1.74 ± 0.10 ɳg/mL	[215]
Tomatoes	Basque Country	6.8 to 23.0 μg/g	[227]
Watermelon	Greece Roamania	0.95 μg/g 0.90 μg/g	[212]
White bread	Egypt	3.20 ± 0.36 μg/g	[215]
Wild chive	South Korea	3.06 ± 0.04 μg/g	[216]
Wild parsley	South Korea	3.45 ± 0.05 μg/g	[216]
Wine	Greece	2.2 to 10 mg/L	[228]

Content expressed as interval, standard deviation (SD) or parts per million (ppm)

**Table 2:** Summary of Mn adequate intake ages 0 through 18 years

Gender	Age group	AI (mg/day of Mn)
–	0–6 months	0.003
	7–12 months	0.6
	1–3 years	1.2
	4–8 years	1.5
Girls	9–13 years	1.6
	14–18 years	1.6
Boys	9–13 years	1.9
	14–18 years	2.2

Source: Institute of Medicine’s Dietary Reference Intake for Mn

*Abbreviations*: *AI* adequate intake

The physiological concentration of Mn in the human brain is estimated to lie between 5.32 and 14.03 ng Mn/mg protein (20.0–52.8 μM Mn), whereas 15.96–42.09 ng Mn/mg protein (60.1–158.4 μM Mn) is the estimated pathophysiological threshold [5]. Mn is essential for several physiological processes participating in enzymatic reactions as a cofactor. Mn acts in gluconeogenesis as an activator of pyruvate carboxylase and in the Krebs Cycle as a cofactor for isocitrate dehydrogenase. In the antioxidant defense system, Mn is part of superoxide dismutase (SOD). Moreover, Mn is present in the central nervous system (CNS) as a cofactor for glutamine synthetase (GS), which is preferentially localized in astrocytes [6]. Mn deficiency is a rare concern. Few reports of experimental Mn deficiency have cited poor bone growth, skeletal abnormalities, ataxia, skin alterations and hypocholesterolemia [4, 7].

Mn overload may arise from an impaired or not fully developed excretion system, transporter malfunction or exposure to excessive levels of Mn by air, water, food or total parenteral nutrition (TPN). Given the similarities between Mn and iron (Fe), homeostasis of both metals is interdependent, thus the Fe status also influences Mn accumulation. This is noted in cases of anemia, for example, when low levels of Fe facilitate Mn uptake [8]. Occupational exposure is one of the main concerns for Mn intoxication and it occurs in activities involving mining, welding, battery manufacture and with the use of fungicides containing the metal in its composition, such as maneb and mancozeb [9–12]. Periods of occupational exposure of 6 months to 2 years may lead to the development of manganism. The motor and neuropsychiatric symptoms may remain even 14 years after the end of exposure to Mn [13].

The risk of Mn exposure is not limited to miners or welders. The availability of the metal in the environment, water or food containing high levels of Mn represents a source of contamination for the general population [14]. Furthermore, the levels of Mn in the atmosphere may increase secondary to the use of the gasoline additive methylcyclopentadienyl manganese tricarbonyl (MMT) [15]. Drug abuse has recently become a concern for Mn poisoning, since abusers of the injectable drug methcathinone may be exposed to contaminating Mn due to the use of potassium permanganate in the synthesis process [16]. Patients with hepatic impairment and those receiving TPN, especially newborns, are susceptible to Mn accumulation [9, 17–19]. Infants and children are particularly vulnerable to inappropriate supplementation of Mn, which in some cases may lead to hypermanganesemia, dependent upon the duration of the treatment [17, 18, 20, 21]. Additionally, Mn is present at levels considered excessive in children’s formula [17].

Mutations in the SLC30A10 gene have been reported to induce a genetic Mn overload syndrome. SLC30A10 is a Mn transporter and a recessive loss-of-function mutation in its gene causes a syndrome of movement disorder and chronic liver disease. Magnetic resonance imaging (MRI) of patients with this mutation shows Mn accumulation in the basal ganglia and white matter, even in the absence of previous exposure to high Mn levels [3, 22, 23].

The central nervous system (CNS) is the main target of Mn. Excess Mn preferentially accumulates in the basal ganglia, especially in the striatum (caudate nucleus, *putamen* and *nucleus accumbens*), *globus pallidus* (GP) and the *substantia nigra* (SN) [24, 25]. Recently, the SN pars compacta (SNpc) was identified as a site of Mn accumulation in rats exposed intraperitoneally (ip) [26]. The neurodegenerative process induced by accumulation of Mn is called manganism. Manganism is a syndrome similar to Parkinson’s disease (PD), characterized by psychiatric and cognitive deficits and motor impairment [27, 28]. Mn is also a putative environmental modifier of Huntington’s disease (HD) [29–31]. The symptoms caused by the accumulation of Mn include dystonia, bradykinesia and rigidity due to damage to dopaminergic (DAergic) neurons and gliosis [12, 32]. Manganism and PD affect different areas of the brain, which allowing for a distinction between the two syndromes. SNpc DAergic neurons are progressively lost in PD, whereas the GP is predominantly affected in manganism. Lewy bodies formation is a hallmark of PD, which is not observed in manganism. In addition, manganism is not responsive to treatment with DA precursor levodopa, a drug used in the early stages of PD. Furthermore, manganism presents with a lack of resting tremor but consistent presence of dystonia [33–35].

Mn exposure alters intracellular signaling pathways in mouse and rat striatum, as well as cell culture models. These include alterations in Akt, ERK, p38, DARPP-32 and tyrosine hydroxylase (TH) phosphorylation [36–42]. Transcription factors’ localization, such as NF-κB and NF*-*E2*-*related factor 2 (Nrf2), is affected [43, 44]. Of particular interest, Mn-induced p53 phosphorylation, as well as upregulation of p53 levels, have been shown to be important events in cellular response to Mn exposure both in vivo and in vitro, possibly contributing to neuronal apoptosis [31, 45–47]. Endoplasmic reticulum (ER) stress is another factor that may lead to Mn-induced apoptosis [48].

A proper balance of Mn levels is essential for maintenance of health and avoidance of neurotoxicity. It is thus imperative to study the regulatory mechanisms of Mn uptake as well as its molecular mechanism of toxicity. The main topics of this review will focus on Mn effects in the brain, especially mechanisms of Mn transport and disruption of neurotransmitter signaling. We will discuss the behavioral aspects of Mn intoxication and possible neuroprotective strategies.

## Main text

### Mechanisms of Mn uptake into the CNS

As Mn is required for multiple cellular events but becomes toxic at high levels, the intracellular Mn concentration has to be under strict control. Several mechanisms regulate Mn homeostasis in the CNS, which mainly relies on different Mn transporters. Given the similar physical properties of Fe and Mn, most transporters are able to transport both metals, which compete for binding at the plasma membrane. To date, no proteins are identified as Mn-specific transporters. The brain is protected by the blood-brain barrier (BBB) and there are primarily two ways for Mn to cross the BBB and reach the brain for its function, discussed below.

#### Membrane localized Mn importers

Membrane importers are the primary route of Mn transport into the CNS. These transporters include the divalent metal transporter 1 (DMT1), Zrt-like, Irt-like proteins ZIP8 (SLC39A8) and ZIP14 (SLC39A14), dopamine transporter (DAT), voltage-regulated, store-operated and ionotropic glutamate receptor Ca channels, choline transporters and the citrate transporter [49, 50]. These proteins are localized on cell membranes and are able to form a membrane pore to take up divalent Mn from the extracellular matrix. Moreover, Mn may block transient receptor potential channel (TRPC3), a receptor-operated plasma membrane channel of astrocytes that responds to ATP-induced Ca signaling, thus decreasing purinergic signaling [51].

DMT1 is the most representative and best studied one. It is also known as divalent cation transporter 1 (DCT1), natural resistance-associated macrophage protein 2 (NRAMP 2) or solute carrier family 11 member 2 (SLC11A2). Gunshin et al. (1997), first cloned and characterized DMT1 with a wide range of substrates, including Fe^2+^, Zn^2+^, Mn^2+^, Cu^2+^, Co^2+^, Cd^2+^, Ni^2+^ and Pb^2+^ [52]. Garrick et al. (2006), showed that Mn is DMT1 preferred substrate, with the following transport affinity (reflecting transport efficacy): Mn > Cd > Fe > Pb ~ Co ~ Ni > Zn [53]. Thus, although Fe has also been linked to PD pathology, Mn might play a more prominent role in this disease given its higher affinity for DMT1. In the brain, DMT1 is highly expressed in the basal ganglia, including SN, GP, hypothalamic nucleus and striatum [54–56], rendering these regions more susceptible to Mn accumulation and toxicity. DMT1 regulates Mn influx into neurons by two ways. One is via a direct transport mechanism whereby the membrane-localized DMT1 opens up a pore and allows the extracellular divalent Mn to enter neurons. The other way is via a transferrin (Tf)-dependent process, which will be discussed next.

#### Transferrin (Tf) and transferrin receptor (TfR)

While the majority of Mn in the body is in the divalent oxidation state, there is a small amount of trivalent Mn, which is not a substrate for the above referenced importers. Tf/TfR facilitates Mn^3+^ influx into the CNS from the blood stream [57]. Tf is synthesized in the liver and then released into the blood [58]. Mn exposure increases the expression of TfR by enhancing the binding of iron regulatory proteins (IRPs) to iron responsive element-containing RNA in vitro [59]. TfR is a membrane protein with high affinity for Mn, which is expressed in neurons, microglia, astrocytes and the endothelial cells of the BBB [60]. When TfR recognizes and binds to Tf, the cell membrane expands inwardly and forms an endocytic vesicle, which brings in the Mn [67, 74]. Mn^3+^ is a stronger oxidizing agent than Mn^2+^ and it may cause severe oxidative stress. Ferrireductase reduces Mn^3+^ into Mn^2+^, which is released into the cytosol by DMT1 localized on the endosomal membrane [50].

#### Mn export in the CNS

Efflux plays a fundamental role in regulating intracellular concentrations of Mn in the CNS. Compared with Mn import, efflux of Mn is less studied, partially due to limited proteins identified in Mn export. However, with the recent discovery of four proteins facilitating Mn export, the role of Mn export has begun to be elucidated. These four proteins include ferroportin (Fpn), SLC30A10 (solute carrier family 30 member 10), secretory pathway Ca^2+^-ATPase 1 (SPCA1) and ATPase 13A2 (ATP13A2 or PARK9). Among them, Fpn and SLC30A10 are able to directly export cytosolic Mn out of neurons, while SPCA1 and ATP13A2 indirectly regulate Mn efflux through the Golgi apparatus and lysosome, respectively. Together, these proteins maintain Mn homeostasis in the CNS and mutations in them have been associated with certain diseases.

#### Membrane localized Mn exporters

Currently, these exporters include Fpn and SCL30A10. Fpn was the first known Mn exporter, however, it was first identified as a Fe exporter. And that is why it is also known as iron-regulated transporter 1 or solute carrier family 40 member 1 (SLC40A1). In the brain, Fpn has been found in neurons, astrocytes, the endothelial cells of the BBB, oligodendrocytes, the choroid plexus and ependymal cells [61]. Fpn expression levels are increased in mice and human embryonic kidney cells in the presence of Mn [62]. *Xenopus laevis* oocytes expressing human Fpn showed lower intracellular Mn and higher extracellular Mn [63]. Although these results indicate Fpn may play an important role on Mn homeostasis in the CNS, a direct study to investigate brain Mn levels in human or animal models carrying Fpn mutations has not been reported yet.

Interestingly, the recently identified SLC30A10 has been well known to play a critical role in regulating CNS Mn homeostasis. Currently, it is the only known protein associated with the first hereditary or familial form of Mn-induced parkinsonism. People carrying mutations in SLC30A10 suffer from hypermanganesemia with dystonia, polycythemia and hepatic cirrhosis [22, 64, 65]. The patients have ~10-fold increase in blood Mn levels and magnetic resonance imaging (MRI) studies show high levels of Mn accumulated in the basal ganglia without a history of exposure to elevated Mn from environmental or occupational sources [66]. The mechanisms by which mutations in SLC30A10 mediate Mn accumulation were recently characterized in rat-derived differentiated γ-aminobutyric acid (GABA) ergic AF5 cells, primary mice midbrain neurons and *C. elegans*. Leyva-Illades, Chen et al. (2014), found that wild type (WT) SLC30A10 is localized on the cell membrane, while 5 mutant transporters are all trapped in the endoplasmic reticulum (ER) or in the cytoplasm [67]. While the WT protein is able to protect from Mn induced DAergic neurodegeneration and cell toxicity, the mislocalization deprives these mutants of this essential efflux with ensuing retention of high Mn concentrations in the plasma.

#### Mn efflux mediated by SPCA1 and ATP13A2

SPCA1 is a Golgi-localized Ca/Mn ion pump, which belongs to the P-type ATPase family, with highest expression in keratinocytes but also in other tissues including liver and brain [68]. In HeLa cells, SPCA1 is required for transport of Mn into the Golgi, followed by secretion via exocytosis as a *bona-fide* Mn efflux pathway [69].

ATP13A2 (PARK9) is a transmembrane cation transporting ATPase localized on the membrane of vacuoles and lysosomes [67]. ATP13A2 has been associated with early-onset parkinsonism and Kufor-Rakeb syndrome [70–72]. In primary rat neurons, ATP13A2 levels were increased in the presence of excess Mn, while expression of wild-type ATP13A2 lowered intracellular Mn levels and prevented Mn-induced neuronal death [73].

Despite the evidence in cell culture studies, the role of SPCA1 and ATP13A2 in mediating Mn efflux in the CNS remains unclear. MRI studies to investigate Mn accumulation in the brain of patients or animal models carrying mutations in SPCA1 or ATP13A2 are needed to validate the results from the cell culture studies. The most studied Mn importers and exporters are summarized in Table 3.

**Table 3:** Transporters and their roles in Mn uptake and efflux

	Transporter	Localization	Role in Mn transport	Associated pathologies	Reference
Mn importers	DMT-1	Highly expressed in the basal ganglia	Transports both Mn and Fe and a range of other cations.	Increased expression of DMT1 has been found in the SNpc of PD patients. Alterations in DMT1 are associated with spinal onset amyotrophic lateral sclerosis, AD onset in males, iron anaemia and restless legs syndrome	[3, 50]
	ZIP8 and ZIP14	Apical surface of various cell types	Regulation of Mn homeostasis (in duodenum, liver, brain, lungs, and kidney) and transfer of Mn, Fe, Zn, and Cd into the cells	ZIP 8 and −14 facilitate Cd accumulation, a non-essential toxic metal. MT-null Cd-resistant cells have been found to exhibit suppressed expression of both ZIP8 and ZIP14, suggesting that the down-regulation of both contributes to the decrease in Cd and Mn uptake	[229–231]
	DAT	Neurons of SNpc, GP and striatum	Reuptake of dopamine into presynaptic vesicles. Also shown to transport Mn	Patients chronically exposed to Mn display decreased DAT density and activity. DAT knockout mice exposed to Mn accumulate significantly less Mn in the striatum compared to WT	[50, 232]
	Ca channels	Plasma membrane	Voltage-regulated, store-operated Ca^2+^ channels as well as ionotropic glutamate receptors also facilitate Mn uptake into the brain	The number of known ion channel diseases (channelopathies) has increased dramatically and include cystic fibrosis, Bartter syndrome and epilepsy	[233, 234]
	Choline transporter	Plasma membrane	Choline uptake was found to be significantly inhibited in the presence of Cd and Mn, but not Cu or Al	Prenatal choline deficiency is associated with increased choline transporter mRNA expression in the septum and hippocampus of rats as a compensatory mechanism for acetylcholine synthesis	[235, 236]
	Citrate transporter	Plasma membrane	Mn citrate represents the major non-protein-bound species of Mn to enter the brain at the BBB. The influx transfer coefficient for Mn citrate was shown to be greater than that of Mn^2 +^ alone and Tf–Mn^3+^	Defects in SLC25A1, a mitochondrial citrate carrier, were identified to cause combined D-2- and L-2-hydroxyglutaric aciduria	[237, 238]
	Tf/TfR	Tf in plasma and TfR in the membrane of neurons, microglia, astrocytes and the endothelial cells of the BBB	Tf/TfR facilitates Mn^3+^ influx into the CNS from the blood stream	Polymorphisms in TfR gene have been correlated with increased risk of age related macular degeneration (AMD)	[57, 237, 239, 240]
Mn exporters	Fpn	Transmembrane, expressed in the duodenum, liver, spleen, intestine, endothelial cells of the BBB, neurons, oligodendrocytes, astrocytes, choroid plexus and ependymal cells	Increased Fpn expression in HEK293 cells is associated with decreased intracellular Mn concentration and attenuated cytotoxicity	Mutations in *Fpn* cause type IV hemochromatosis, commonly known as Fpn disease, characterized by Fe accumulation in reticuloendothelial macrophages	[50, 62, 63]
	SLC30A10	Cell surface-localized. Present in basal ganglia and liver	Mediates Mn efflux from cells	Mutations in *SLC30A10* that impair Mn export induce hypermanganesemia, dystonia, and polycythemia with a variable degree of hepatic dysfunction	[22, 23]
	SPCA1	Mainly in Golgi membrane of keratinocytes, liver and brain	Imports Mn^2+^ from the cytosol to the Golgi lumen	Monoallelic mutations in *SPCA1* are known to cause Hailey-Hailey disease, a blistering skin disorder. Complete loss of function is thought to be unviable	[3, 241]
	ATP13A2 or PARK9	Transmembrane, localized on the membrane of vacuoles and lysosomes	Cation transporting ATPase. Shuttles cations across lysosomal membrane	ATP13A2 mutations have been associated with early-onset parkinsonism and Kufor-Rakeb syndrome.	[242–244]

*Abbreviations*: *AD* Alzheimer’s disease, *ATP13A2* ATPase type 13A2, *DAT* dopamine transporter, *DMT1* divalent metal transporter 1, *Fpn* ferroportin, *MT*, metalothionein, *SLC* solute carrier, *SPCA1* secretory pathway Ca2 + −ATPase isoform 1, *Tf* transferrin, *TfR* transferrin receptor

Recently, a high throughput screening approach was carried out to identify small molecules responsible for intracellular regulation of Mn homeostasis at physiologically relevant levels. It’s suggested that intracellular Mn levels are actively controlled by the cell and not exclusively by the BBB or blood-cerebrospinal fluid barrier. Furthermore, mechanisms regulating Mn content might be developmentally regulated in DAergic neurons reflecting the changing physiological demand [74].

### Mn and the cholinergic system

Mn-induced alterations in behavioral patterns, namely motor incoordination or emotional and cognitive dysfunction, which observed in both patients and/or animal models, are associated with neurotransmitter metabolism disruption. Impaired neurotransmitter signaling may occur via diverse mechanisms, such as neurotransmitter release inhibition, alterations in neurotransmitter clearance from the synaptic cleft, or modulation of receptor levels or activity. The main neurotransmitter system studied in Mn neurotoxicity is the dopaminergic (DAergic) system [24, 75]; several studies have also described Mn’s effects on the GABAergic [76] and glutamatergic systems [77–82].

Mn at neurotoxic levels also affects the cholinergic system. Acetylcholine (ACh) is an important excitatory neurotransmitter both in the central and peripheral nervous system, modulating essential cognitive functions, such as learning, memory and locomotion. Given the scarcity of attention devoted to this system, we will focus next on Mn’s effects and cholinergic dysfunction [83–86].

The cholinergic system encompasses the neurotransmitter ACh, the enzyme that synthesizes ACh named Choline Acetyltransferase (ChaT; E.C. 2.3.1.6), the enzymes that hydrolyze ACh called cholinesterases (acetylcholinesterase-AChE; E.C. 3.1.1.7 and butyrylcholinesterase-BuChE; E.C. 3.1.1.8), by the cholinergic receptors (muscarinic and nicotinic) and by the system that reuptakes choline. Dysfunction of the cholinergic system is associated with several diseases, such as Alzheimer’s disease (AD) and *myasthenia gravis*. Mn effects on the cholinergic system may contribute to impairments in learning, memory and locomotion [87]. Although several symptoms of PD and manganism are largely related to effects on the DAergic system, studies suggest that the cholinergic system might play an important role in such diseases [83, 87]. Furthermore, Mn’s toxic effects might be related to an imbalance between the DAergic and cholinergic systems, predominantly in the basal ganglia [83].

ChAT is a marker of cholinergic function. A decrease in its activity leads to diminished storage and release of ACh affecting directly its function. Several reports have addressed the ability of Mn to alter ChAT activity. Numerous factors may contribute to this effect, including the age of the animals and the duration of treatment, since cholinergic neurons are exquisitely vulnerable in the developing brains [83, 84].

AChE is an important regulatory enzyme that rapidly hydrolyzes ACh at brain cholinergic synapses as well as at the neuromuscular junction [88, 89]. AChE possesses unique characteristics not found in any other enzyme, such as its active site organization and its catalytic mechanism [90–92]. AChE is extremely important in regulating brain function, development, neurite outgrowth, neuronal survival, and calcium levels [83, 93]. Various toxicological conditions that generate oxidative stress alter AChE activity, mainly its membrane bound form. Such changes in activity are commonly accompanied by clear signs of neurobehavioral alterations [83, 94, 95]. For example, an increase in the enzyme activity was observed by [95] and [96] correlating positively with thiobarbituric acid reactive substances (TBARS) production, possibly due to lipid peroxidation.

Several studies have addressed Mn’s influence on AChE activity. Table 4 summarizes the source of the enzyme and Mn’s effect on its activity. It is important to emphasize that Mn effects in biological systems depend on the routes of exposure, dose, age, period of exposure, environmental factors and nutritional state [83, 87, 94, 97–100].

**Table 4:** Effects of Manganese (Mn) exposure on AChE activity in different experimental protocols

Model	Tissue	Administration route and dose	Effect on AChE activity	Reference
Adult rats	Whole Brain	Intraperitoneal-acute (10–15 mg/kg) and chronic (mg/kg)	Increase	[100]
Adult rats	Cerebellum	Oral via by drinking water, 30 days (20 mg/ml)	Increase	[189]
Rats from 21–74 days	Brain	Oral via by food, for 53 days, chronic exposure (500 mg/kg)	Increase	[94]
Adult mice	Hypothalamus, pons, cerebellum, striatum, medulla, cerebral cortex and hippocampus.	Chronic treatment by oral via from conception to 60 days old	No alterations	[98]
0-adult rats	Cerebellum and striatum	Oral via by drinking water, 60 days (20 mg/ml)	Increase	[97]
Adult rats	Brain	Intraperitoneal, 7 days (50 mg/kg)	Increase	[99]
Adult rats	Whole Brain	Intraperitoneal-(25 mg/kg) in 4 and 8 doses	Decrease	[84]

**Fig. 1:**
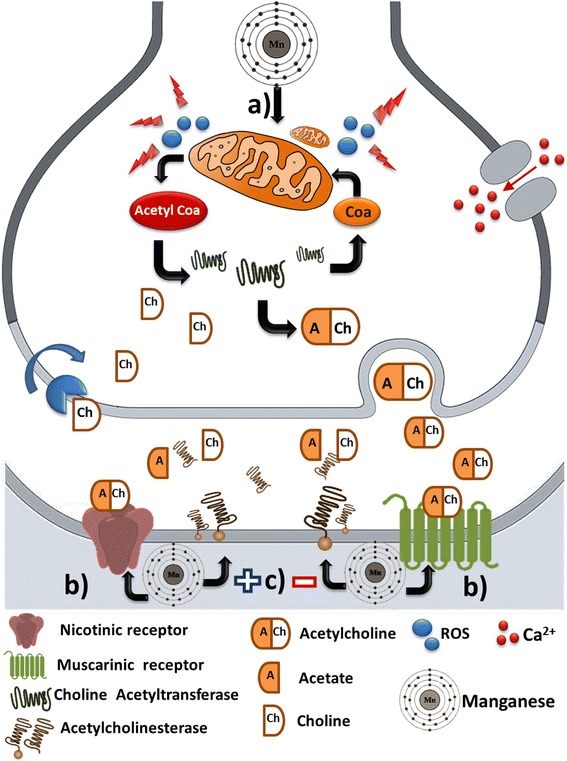
Overview of Manganese (Mn) effects on cholinergic signaling. **a** Mn promotes an increase in reactive oxygen species production through of mitochondrial dysfunction. In addition, Mn impairs the synthesis of precursors for acetylcholine neurotransmitter production. **b** Mn induces up-regulation of nicotinic and muscarinic receptors. **c** Mn has a controversial effect on acetylcholinesterase since it is able to increase, reduce or not alter the activity of this enzyme ﻿across diferent models of Mn exposure﻿

ACh binds to two types of cholinergic receptors: the ionotropic family of nicotinic receptors and the metabotropic family of muscarinic receptors. The nicotinic acetylcholine receptor (nAChR), at the nerve/muscle synapse, is one of the best-characterized transmitter-gated ion channels [101, 102]. The muscarinic receptors belong to the large superfamily of plasma membrane-bound G protein coupled receptors (GPCRs) [103]. The muscarinic receptor family has five known members designated M1–M5. Mn exposure can affect the binding of ACh to cholinergic receptors. For example, intranasal Mn treatment in adult mice down-regulates nicotinic acetylcholine receptors (nAChR) in the prefrontal cortex in wild-type (high Fe accumulation) Hfe^+/+^ and Hfe-knockout Hfe^−/−^animals [85]. However, in other studies no alterations were found in the binding or density of cholinergic receptors. Chronic administration of MnCl_2_ (5 mg Mn/kg body weight/day) for 9 weeks, did not affect the [^3^H]-quinuclidinyl benzilate binding to muscarinic cholinergic receptors in mouse brain [104]. No changes in the muscarinic receptor density (B_max_) and the dissociation constant (K_d_) of 3H-QNB in the different mouse brain regions was observed after daily ip injections of MnCl_2_ (5 mg Mn/kg) for 9 weeks [105]. Finally, the density of muscarinic receptors in monkeys remained unchanged after Mn exposure for 26 months at a dose comparable to what workers might inhale in dusty environments [106]. An overview of the potential effects of Mn on cholinergic function is depicted in Fig. 1.

Cholinergic signaling is involved in anti-inflammatory reactions. ACh is the main vagus neurotransmitter [107–109] and the efferent arm of the inflammatory reflex, now termed the cholinergic anti-inflammatory pathway. It is a highly robust mechanism for cytokine control [110]. The vagus nerve releases ACh when stimulated (either electrically or pharmacologically), inhibiting macrophage activation and release of pro-inflammatory cytokines, e.g. interleukin-6 (IL-6), tumor necrosis factor alpha (TNF-α), IL-1 and IL-18. One of the molecular mechanisms for cytokine synthesis inhibition is attributable to ACh [107, 108, 111, 112]. Accordingly, the cholinergic system controls inflammatory process and is recognized as a possible marker of low-level systemic inflammation [113–115].

### Behavioral consequences of Mn exposure in humans and experimental models

#### Mn exposure by inhalation in occupational settings

It is estimated that over one million workers in the USA perform welding as part of their job. The pipes used in heating and ventilation systems as well as industrial process piping often require welding, which is also essential for ductwork, laboratory hoods, tanks, boilers, and process vessels. Welding produces respirable fumes that may contain Mn as well as other chemicals, such as chromium, arsenic, iron and nickel. The level of Mn exposure varies dependent upon the type of welding activity performed, ranging from 0.01 to 2.0 mg/m^3^ [116]. In contrast, the world health organization (WHO) recommends that levels of Mn do not exceed 30 μg/m^3^. It has been demonstrated that the use of ventilation systems reduces these values and could be an effective approach to minimize Mn exposure [116].

Using rats to model Mn exposure via inhalation, it has been demonstrated that the inhalation route is more efficient than ingestion at delivering Mn to the brain [117]. Mn is taken up via the olfactory tract and transferred along olfactory neurons processes through the cribriform plate to synaptic junctions with olfactory bulb neurons, thus bypassing the BBB. Once in the brain, Mn can continue to traverse synapses and be transported along neuronal tracts to other sites of the brain [118, 119]. Furthermore, the accumulation of Mn in the blood after intranasal instillation is much greater than via the oral route because Mn bypasses the biliary excretion [120]. DMT-1 is important for Mn transport across the olfactory epithelium into the brain of rats and can be influenced by Fe status [121]. Other transporters may regulate Mn uptake from the olfactory epithelium. Candidates are SLC30A10 or Mn binding proteins [120]. DMT-1 also plays a role in lung uptake of inhaled Mn [122].

Several studies point to a strong correlation between occupational Mn exposure and an increased risk of PD [123]. Parkinsonian symptoms in welders attributed to Mn exposure have been reported in numerous studies. A statistically significant difference in the age of PD onset between welders (46 years of age) and a control group (63 years) [124] has been noted. Alpha-synuclein (α-Syn), the major component of Lewy bodies and the hallmark of PD, contains metal binding sites, and its activity is not yet fully understood. It has been proposed that α-Syn attenuates Mn-induced DAergic degeneration in the early stage, but after prolonged exposure, Mn promotes α-Syn aggregation [125]. In *C. elegans*, α-Syn attenuates Mn-induced toxicity in the background of PD-associated genes [126]. Recently, α-Syn has been proposed to act as a intracellular Mn store [127].

Because of its paramagnetic properties, Mn accumulation can be visualized using T1-weighted magnetic resonance imaging (MRI) [128]. In a study of 193 subjects exposed to welding activities from the Midwestern USA it was shown that Mn accumulates throughout the basal ganglia, with a diffused T1 signal as well as elevated blood Mn levels when compared to age and gender matched controls. However, it was found that the MRI data not always correlated with clinical symptomatology [129, 130]. This may occur because modern occupational exposure to Mn occurs at much lower levels than reported in the past, resulting in a less distinguishable clinical phonotype. Even asymptomatic welder apprentices display increased T1 signal in the basal ganglia, but when evaluated in the Grooved Pegboard (for dexterity and fine motor control) or the unified PD rating scale motor subsection 3 (UPDRS3-for parkinsonian signs such as rest and postural tremor, bradykinesia and gait disturbance), the subjects performed within the reference range [131]. Nevertheless important neuropathological alterations have been observed even in the absence of motor symptoms [129, 132, 133]. It is not clear from the clinical studies, however, whether Mn facilitates the development of PD or induces a distinct parkinsonian syndrome. Future studies should address this issue by clearly diagnosing either PD or manganism based on the known distinctions between the two diseases.

To better understand the significance of MRI findings, an ex vivo study correlated imaging with neuropathology in 19 mine workers and 10 race- and sex-matched controls from South Africa (where 80 % of the world’s Mn reserves are located). It was found an inverse relationship between the T1 intensity indices and neuronal density in the caudate and putamen, suggesting neuronal loss. The authors also noted increased microglial cell density in the basal ganglia. Based on this and their previous study [133] they propose that the pre-clinical stage of Mn-induced neurotoxicity is marked by an initial inflammatory response that may progress to astrocyte isruption and neuronal injury [132]. This would be in agreement with in vitro findings that report a 50 fold higher accumulation of Mn in astrocytes, which may alter their neurotrophic actions and contribute no neuronal injury [134–137]. Astrocytes are initially affected in manganism showing changes in the expression of glial fibrilary acidic protein (GFAP) preceding neuronal death [138]. Increased GFAP expression is observed in the striatum of rats, which indicates glial activation in response to Mn [139, 140]. Microglial cells are also affected by Mn with increased release of proinflammatory cytokines [134] and may activate astrocytes to release inflammatory mediators such as prostaglandin E2 and nitric oxide [141].

#### Environmental Mn exposure

Contaminated air or water pose a risk of Mn intoxication to the general population. Mn exposure from environmental sources has also been associated with a higher prevalence of Parkinsonian disturbances [142]. For instance, near foundries, Mn concentrations may reach 200–300 ng/m^3^, contrasting to normal levels of Mn in the air that are around 10–30 ng/m^3^ according to the WHO. Recently, a study by Bowler et al. (2015) was performed to assess cognitive function in adults environmentally exposed to Mn in Ohio, USA, in two towns identified as having high levels of air-Mn from industrial sources. The authors report that non-occupational environmental Mn exposure appears to be associated with lower performance on neuropsychological tests measuring a variety of cognitive functions [143].

North America’s longest operating ferromanganese refinery is located in Marietta, Ohio, USA. To address the population leading environmental public health concern, a study was conducted to evaluate children’s cognitive function. It was found that both high and low blood and hair levels of Mn could negatively impact children’s IQ, consistent with the notion that Mn is both a nutrient and a neurotoxicant. Of note, lead (Pb) and cotinine (a nicotine metabolite) were also measured in children’s blood, serum or hair since environmental exposures to toxic chemicals rarely occur isolated. Pb levels in blood of that study population were similar to mean blood Pb of children in the USA and did not influence IQ scores. Cotinine levels were significantly associated with IQ scores, demonstrating that secondhand tobacco smoke can negatively impact child cognitive function [144]. Airborne Mn also detrimentally influenced children’s postural stability in this population [145]. Mn has been identified as a developmental neurotoxicant associated with hyperactivity, lower intellectual function, impaired motor skills and reduced olfactory function in children [146, 147]. In animal models, the immature CNS is more susceptible to Mn neurotoxicity compared to the adult [148] and experimental evidence suggests that exposure to this metal during development can affect neurological function in adulthood [139, 140, 149, 150].

The presence of excessive Mn levels in drinking water has been associated with poorer memory and attention [14], and hyperactive behavior [151] in school-aged children. Consumption of water containing elevated Mn levels had adverse effects on 10-year-old children’s cognitive function [152]. Children exposed to elevated airborne Mn in an area close to a ferromanganese alloy plant in Brazil presented lower I.Q., impairment of verbal skills [153] and lower neuropsychological performance in tests of executive function of inhibition responses, strategic visual formation and verbal working memory [154].

#### Mn and parenteral nutrition

Mn is present in parenteral nutrition formulations both as an essential element but also as a contaminant, thus posing as an important source of excessive exposure to Mn. The content of Mn in TPN varies from 0.18 μmol/d (0.01 mg/d) to 40 μmol/d (2.2 mg/d) [21]. Toxicity to Mn has been observed in adults receiving >500 μg/d and in pediatric patients receiving >40 μg/kg/d. Furthermore, duration of TPN treatment is associated with increased blood and brain concentrations of Mn [155–157]. Thus, current guidelines recommend monitoring patients for Mn toxicity if they receive TPN longer than 30 days [158].

Parenteral administration bypasses the regulatory mechanisms of the gastrointestinal tract. The bioavailability of Mn in parenteral fluid is 100 %, compared to only 5 % for enteral dietary Mn. For newborns, the Mn burden derived from parenteral nutrition can be 100 times greater than human milk. Of particular importance, the hepatic mechanisms responsible for Mn excretion are not completely developed in newborns. This factor combined with the high bioavailability of the metal in TPN increases the risk of Mn overload. That is also true for patients with hepatic dysfunction [17, 18, 21, 157].

#### Behavioral studies of Mn intoxication

Several reports address the effects of Mn exposure on behavioral tasks [67, 139, 149, 159–170]. Some of these effects are described on Table 5. As for ChAT and AChE activity, it can be observed that the animal model, the duration of exposure and the administration route are important variables when studying behavioral parameters. Briefly, the most common tasks analyzed in the references below are: Morris water maze task (MWM) an hippocampal-dependent learning test, including acquisition of spatial memory and long-term spatial memory [171]; 8-arm radial maze paradigms to evaluate reference and working memory performance simultaneously [172]; active avoidance paradigms that utilizes the passive avoidance and active avoidance test paradigms, which assay different forms of fear-based conditioned avoidance considered to be an escape response [173]; variable delayed response (VDR) task where monkeys are trained to perform cognitive tasks while seated in a restraining chair. VDR analyzes both attentional and spatial working memory components [165]; self-ordered spatial search (SOSS) task and Five Choice serial reaction time (5-CSRT) task. The SOSS task requires animals to touch identical squares located in different spatial locations in a self-ordered sequence without returning to a previously touched square. The 5-choice serial reaction time (5-CSRT) task is a widely used test to measure multiple aspects of cognition including attention, impulsivity and perseveration [167]; The object recognition task utilizes the exploration time spent in the new and familiar objects are used as parameters to asses memory and finally the social recognition test to observe short-term memory impairments [139].

**Table 5:** Effects of Manganese (Mn) on different behavioral tasks

Experimental model	Treatment	Behavioral task	Results	Reference
Male Sprague-Dawley rats	Neonate rats were orally exposed to MnCl_2_(0, 25, 50 mg/kg/day) between PND1-21	8-arm radial maze paradigm	Mn-exposed males showed working memory impairment	[149]
Young adult male Wistar rats	One group was treated with 14.84 (low dose group), and another one with 59.36 (high dose group), mg/kg Mn given by gavage, 5 days a week for 10 weeks.	8-arm radial maze task	MnCl_2_ treated groups showed, compared to control animals, a decrease in the average memory performance	[170]
Male Wistar rats	Single oral doses of MnCl_2_ (50 mg/kg) or chronic oral MnCl_2_ (20 or 50 mg/kg/ day) for 1 month	Active avoidance paradigm	Single dose induced decline of the memory acquisition of an avoidance reaction in response to unconditioned and conditioned stimuli. Chronic manganese poisoning also led to significant impairment of learning processes	[168]
*C. elegans*	1 h Mn (10 or 25 mM) exposure on L1 larval stage	Basal slowing response	Mn-exposed worms had an impaired basal slowing response, indicating DAergic damage. This was reversed by SLC30A10 (a cell surface-localized Mn efflux transporter) expression in DAergic neurons	[67]
*C. elegans*	30 min Mn (50 mM) exposure on L1 larval stage	Dauer movement	In WT dauer worms, the locomotion was increased in the presence of Mn, indicating DA signaling impairment	[161]
Male and female Sprague-Dawley rats	Pregnant females treated with Mn (2 mg/ml) in drinking water from the first day of pregnancy until PND20.	MWM	PND 21–25: Mn-exposed females displayed memory deficits in the probe trial PND 56–60: Mn-exposed males performed significantly worse in the acquisition trial. Females exposed to Mn displayed deficits in learning and memory	[159]
Three-month-old male Wistar rats	Intranasal 2-week-long MnCl_2_ (0.8 mg/kg body weight)	MWM	Spatial memory deficits	[160]
Male Sprague-Dawley rats	Intraperitoneal injection of MnCl_2_ 15 mg/kg for 8, 12 or 18 weeks	MWM	Escaping latency and swimming distance of rats in the model groups increased, suggesting spatial learning and memory impairment	[162]
Male Sprague Dawley rats	Intraperitoneal injections of 0, 5, 10 and 20 mg/kg MnCl_2_ once daily, 5 days/ week for 18 weeks.	MWM	Mn impaired learning and memory as follow: In 6 weeks at dose 20 mg/kg. In 12 weeks at doses 10 and 20 mg/kg. In 18 weeks at doses 5, 10 and 20 mg/kg	[163]
Male Sprague-Dawley rats (6 weeks of age)	Intraperitoneal injections 15 mg/kg MnCl_2_ daily for 8 weeks.	MWM	The escape latency in the Morris water maze test was significantly longer in the rats injected with Mn indicating worsening in spatial memory	[164]
Sprague-Dawley rats, 3-week- old	Intraperitoneal injections (5, 10, 20 mg/kg MnSO_4_) 5 days a week for 24 weeks	MWM	Mn exposure decreased the spatial learning ability in a dose- and time- dependent manner	[169]
Male Wistar rats	Exposed intraperitoneally to MnCl_2_ at doses 5, 10 or 20 mg/kg/day from PND 8–12	Object and social recognition tasks	PND 60–65: Rats exposed to the highest Mn dose failed to recognize a familiar object when replaced by a novel object as well as to recognize a familiar juvenile rat after a short period of time, indicating cognitive impairment	[139]
Adult male *M.* *fascicularis* macaques, 5 to 6 years old	Mn was administered as MnSO_4_ for 15 mg/kg/week for 5 weeks and then 20 mg /kg/week for the remainder of the study period (12 months)	SOSS and 5-CSRT tasks	Deficits in performance of the SOSS task began to appear by the fourth month of Mn exposure but only became consistently significantly impaired beginning at the ninth month of Mn exposure. Performance on the 5-CSRT became significantly affected by the third month of Mn exposure	[167]
Adult male *M.* *fascicularis* monkeys with 5 to 6 years old	MnSO_4_ at doses 10–15 mg/kg/week over an exposure period lasting 272 ± 17 days	Variable delayed response task	Animals developed subtle deficits in spatial working memory and had modest decreases in spontaneous activity and manual dexterity	[166]
Adult male *M.* *fascicularis* macaques, 5 to 6 years old	MnSO_4_ at doses 15–20 mg/kg/week over an exposure period lasting 227.5 ± 17.3 days	Variable delayed response task	Animals developed mild deficits in spatial working memory, more significant deficits in non-spatial working memory and no deficits in reference memory	[165]

*Abbreviations*: *5-CSRT* five choice serial reaction time, *DA* dopamine, *MWM* Morris water maze, *PND* post-natal day, *SOSS* self-ordered spatial search

In *C. elegans*, Mn exposure has been shown to result specifically in DAergic neurodegeneration [174]. In *C. elegans* DAergic neurons are considered mechanosensory and any condition impairing DA signaling will affect the ability to sense or respond to changes in its environment. DA signaling plays an important role in learning and regulation of locomotor behavior, including basal slowing response, ethanol preference, area-restricted searching, habituation task/tap withdrawal response, egg laying, dauer movement, pharyngeal pumping and thrashing behaviors [175, 176]. Among these behaviors, basal slowing response is DA-specific, and other behaviors are usually controlled by DA along with other neurotransmitters, such as serotonin, glutamate or GABA, etc. To date, basal slowing response and dauer movement have been studied with Mn exposure [175, 177, 178]. Levya-Illades, Chen et al. (2014), have shown that Mn exposure resulted in decreased basal slowing response, while expression of Mn exporter SLC30A10 exclusively in DAergic neurons rescued this behavioral defect together with decreased DAergic neurodegeneration [67]. In WT dauer worms, the locomotion was increased in the presence of Mn, indicating DA signaling is damaged by Mn exposure [176]. Similarly, the movement in *djr-1.2* (homolog of mammalian DJ-1) worms was increased, indicating that loss of DJ-1 function resulted in abnormal DAergic neurons.

### Neuroprotective strategies against Mn

Mn-induced neurotoxicity may present in different animal models with distinct damage, depending on time of exposure, dose and route of exposure [179, 180]. In this regard, different therapeutic approaches have been studied in different models. Originally, Mn-induced parkinsonism patients were treated with levodopa, however they were unresponsive to the treatment [181, 182] possibly due to the relatively intact nigrostriatal pathway in the latter phase of the disorder [9]. Hence, other treatments have been tested. We will briefly discuss in vitro and in vivo investigations on the properties of endogenous antioxidants (for instance, vitamin E), plant extracts (complex mixtures containing polyphenols and non-characterized components), Fe chelating agents, precursors of glutathione (GSH), and synthetic compounds that can experimentally afford protection against Mn-induced neurotoxicity.

#### Vitamin E and GSH

Vitamin E and trolox (a hydrophilic analog of vitamin E) have been reported to protect the CNS of rodents and cultured cells from the toxic effects of Mn [183–185]. I.p. exposure of lactating rats to Mn caused striatal and hippocampal oxidative stress and motor impairments, which were prevented by trolox co-administration [183]. GSH and N-Acetylcysteine (NAC), a precursor of GSH, can also decrease the toxicity of Mn in vitro [186]; however, the protective mechanism involved in NAC and GSH has yet to be fully studied. It is likely that these compounds serve as indirect antioxidants since GSH is a substrate of glutathione peroxidase (GPx) enzymes.

#### Plant extracts

Plant extracts have been demonstrated to confer protection against Mn neurotoxicity after in vitro [81] and in vivo exposure in mice [187]. Acai (*Euterpe oleracea)* methanolic extract protected astrocytes from Mn-induced oxidative stress. The protective effects may be associated with the antioxidant and anti-inflammatory effects of its anthocyanin components [81]. Similarly, crude aqueous extracts of *Melissa officinalis* blunted the Mn-induced striatal and hippocampal lipid peroxidation [187]. Purified flavonoids, such as, silymarin (obtained from *Silybum marianum*, a plant with hepatoprotecive properties) protected neuroblastoma cells [188] and prevented Mn-induced oxidative stress in brain, liver, and kidney of rats [189–191]. Lycopene has also been reported to decrease the neurotoxicity of Mn in rats [192].

#### Chelating agents

Because of the chemical similarities between Mn and Fe, it is possible that the neurotoxic effects of Mn might be associated with competition with Fe for “non-redox” domains in proteins [193]. Consequently, compounds with Fe chelating properties or those interfering with the Fenton’s reaction, such as polyphenol compounds, can be of potential pharmacological importance in the treatment of Mn toxicity [194–196]. Indeed, the treatment with a calcium disodium salt of the chelator EDTA (CaNa_2_EDTA) reduced Mn-induced DA autooxidation in vitro [197], enhanced urinary excretion of Mn in humans [198] and reduced Mn levels in the brain and liver of Mn-exposed rats [199]. However, there is still controversy regarding the amelioration provided by this chelating therapy [200, 201].

#### Synthetic compounds

Synthetic molecules have also been reported to reduce Mn toxicity. For instance, several organochalcogens (i.e. organocompounds containing selenium or tellurium atoms bound to carbon) have been reported to possess antioxidant and anti-inflammatory properties [202]. The protective effects of organoselenide and telluride compounds against Mn-induced neurotoxicity, including ebselen, have been reported [184]. One proposed mechanism might be related to a direct scavenger activity against ROS produced by Mn as most of these compounds have thiol-peroxidase activity catalyzed by glutathione-peroxidase isoforms [202]. Using the complementary animal model *C. elegans*, it was shown that these compounds could modulate the transcription factor DAF-16 (FOXO in mammals), increasing its translocation to the nucleus. In turn, the expression of antioxidant enzymes such as superoxide dismutase increased, thus protecting the worms from Mn-induced toxicity [203, 204]. An additional proposed mechanism is the anti-inflammatory action of some of these compounds, e.g. ebselen. Consequently, in addition to counteracting free radicals and modulating gene expression, ebselen and related compounds could decrease Mn toxicity via anti-inflammatory properties. Of note, anti-inflammatory agents have been reported to decrease Mn neurotoxicity in vitro and after in vivo exposure. For instance, Santos et al. (2013) demonstrated in vitro that 5- aminosalicylic acid (5-ASA) and para-aminosalicylic acid (4-PAS) increased mitochondrial and cell viability following Mn exposure [205]. Ibuprofen, a nonsteroidal anti-inflammatory drug, protected striatal neurons from dendritic atrophy and spine loss in rats treated for 2 weeks with the drug prior to Mn exposure [184].

The indirect pro-oxidative effects of Mn have been linked to disruption of synaptic glutamate homeostasis by interfering with glutamate uptake in astrocytes [206]. The increase in extracellular glutamate can cause excitotoxicity, which is associated with oxidative stress in neurons [206]. Furthermore, Mn decreases astrocytic glutamate uptake and expression of the astrocytic glutamate/aspartate transporter (GLAST) via disruption of intracellular signaling [207]. Of potential clinical significance, estrogen and tamoxifen have been reported to increase the expression of glutamate transporters (both GLAST and GLT-1) in astrocytes, potentially decreasing Mn toxicity [77, 207–210]. Raloxifene, which is a selective estrogen receptor modulator, also attenuates the reduction of GLT-1 and GLAST expression and the glutamate uptake induced by Mn in astrocytes [211], thus confirming how promising this class of molecules might be.

Finally, preventing or reducing Mn exposure is essential. For instance, methodologies by which welding fumes generation rate and/or welding practices can be modified to reduce toxic workplace exposures should be sought. In this context, a recent study of Sriram et al. (2015) demonstrated that rats exposed by whole body inhalation to an altered welding process (parameters: voltage, current and shielding gas) showed absence of neurotoxicity when compared to the rats exposed to regular welding process [11]. Reducing Mn levels in infant milk formulas and on parenteral nutrition should also be a strategy as safety policy.

## Conclusions

The interest in researching Mn toxicity has grown in the past few decades. Recent clinical studies in populations exposed to the metal via occupational or environmental sources demonstrate Mn accumulation in the brain with T1-weighted MRI. Evidence for cognitive and motor impairment, especially in children has also been presented. Furthermore, it is evidenced by the work mentioned above that the use of rodent and other complimentary models is an important tool for the study of mechanisms of Mn toxicity, focusing on Mn transport, metal homeostasis, behavioral outcomes and neuroprotective strategies. Animal models facilitate the use of different routes of exposure to Mn, as well as the use of different chemical forms of Mn, that can mimic environmental or occupational exposure. *C. elegans* is also an excellent tool for genetic analysis and manipulations. The availability of mutants and green fluorescent protein (GFP)-tagging makes it easy to explore a wide range of chemicals and their effects. Several effects in response to exposure to metals, especially those involving gene expression and behavior have been reported using the nematode as a model.

One of the particularities of Mn mechanism of action is that it accumulates preferentially in the basal ganglia and targets DAergic neurons. However, various studies show that Mn may also affect other neurotransmitter systems. In this context, it is important to emphasize that to better understand Mn neurotoxic effects a cross talk between DAergic and cholinergic systems seems to be important, specially when regarding the brain regions related with PD and manganism, such as striatum, where cholinergic interneurons are present. Moreover, the neurotransmission at the neuromuscular junction and how it can lead to the motor impairment observed in manganism is an area that needs further exploration.

## Abbreviations

ACh: Acetylcholine
AChE: Acetylcholinesterase
AD: Alzheimer’s disease
AI: Adequate intake
BBB: Blood-brain barrier
ChaT: Choline acetyltransferase
CNS: Central nervous system
DA: Dopamine
DAT: Dopamine transporter
DMT1: Divalent metal transporter 1
FPN: Ferroportin
GABA: γ-aminobutyric acid
GFAP: Glial fibrilary acidic protein
GP: *Globus pallidus*
GPx: Glutathione peroxidase
GS: Glutamine synthetase
GSH: Glutathione
HD: Huntington’s disease
MMT: Methylcyclopentadienyl manganese tricarbonyl
NAC: N-Acetylcysteine
nAChR: Nicotinic acetylcholine receptor
PD: Parkinson’s disease
SN: *Substantia nigra*
SOD: Superoxide dismutase
Tf: Transferrin
TfR: Transferrin receptor
TH: Tyrosine hydroxylase
TPN: Total parenteral nutrition
WT: Wild type
